# Ungewöhnliche Präsentation eines bullösen, multilokulären Erysipels am Rumpf – ein Fallbericht

**DOI:** 10.1007/s00105-025-05635-5

**Published:** 2026-01-08

**Authors:** Chiara L. Blomen, Finn Abeck, Franziska Petersen, Maria Christolouka, Nina Booken, Stefan W. Schneider

**Affiliations:** Klinik und Poliklinik für Dermatologie und Venerologie, Universitätsklinikum Hamburg-Eppendorf, Hamburg, Deutschland

**Keywords:** Erysipel, Bullöses Erysipel, Infektiologie, Differenzialdiagnosen des Erythems, Erysipelas, Bullous erysipelas, Infectiology, Differential diagnosis of erythema

## Abstract

Das Erysipel ist eine akute bakterielle Infektion der Haut und Lymphgefäße, meist durch β‑hämolysierende Streptokokken verursacht. Ungewöhnliche Lokalisationen wie der Rumpf sowie seltene Verlaufsformen, z. B. das bullöse oder multilokuläre Erysipel, erschweren die klinische Diagnosestellung und erfordern die Abgrenzung von Differenzialdiagnosen, wie der Phlegmone, dem SSSS („staphylococcal scalded skin syndrome“) oder der TEN (toxische epidermale Nekrolyse). Bei immunsupprimierten oder onkologisch vorerkrankten Patient:innen sollte auch bei atypischen Erythemen an ein Erysipel gedacht werden.

## Abstract (en)

Erysipelas is an acute bacterial infection of the skin and lymphatic vessels, predominantly caused by *Streptococcus pyogenes*. Atypical localizations such as the trunk, as well as rare clinical variants including bullous or multifocal erysipelas, may complicate the diagnostic process and require differentiation from other blistering or widespread erythematous conditions, such as staphylococcal scalded skin syndrome (SSSS), toxic epidermal necrolysis (TEN) or cellulitis. In immunocompromised or oncologically pretreated patients, erysipelas should be considered even in unusual presentations.

## Anamnese

Ein 47-jähriger Patient stellte sich aufgrund von akut aufgetretenen, progredienten Erythemen unklarer Ätiologie am Rumpf, einhergehend mit Fieber und Abgeschlagenheit, in unserer zentralen Notaufnahme vor.

In der Vorgeschichte war ein nicht-kleinzelliges Bronchialkarzinom bekannt, das mittels Radiatio des pulmonalen Primarius und der mediastinalen Lymphknoten sowie medikamentös behandelt wurde. Vortherapien mit den Zytostatika Carboplatin und Pemetrexed sowie mit dem PD-1-Antikörper Pembrolizumab waren bis vor einem Jahr erfolgt. Die onkologische Erkrankung sei aktuell stabil.

Eine neue Medikamenteneinnahme in den letzten sechs Monaten wurde verneint.

## Befund

**Abb. 1:**
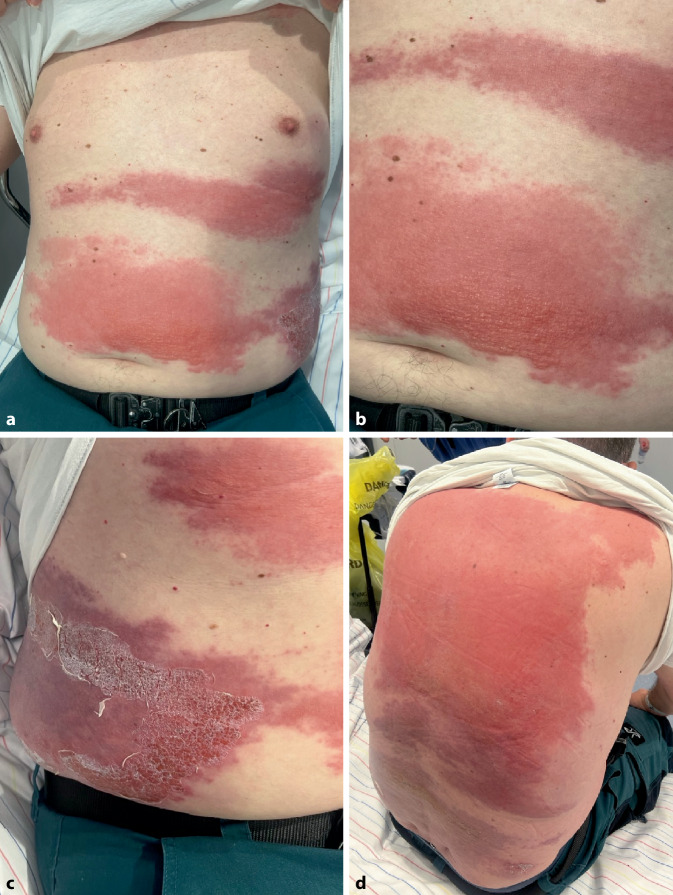
Klinisches Bild bei initialer Vorstellung: multilokuläres Erythem am Rumpf (**a**-**b**: ventralseitig, **c**-**d**: dorsalseitig) mit derb bullöser und squamöser Komponente

Bei Vorstellung präsentierte sich der Patient in reduziertem Allgemeinzustand, begleitet von plötzlich aufgetretenem, undulierendem Fieber und Schüttelfrost seit mehreren Tagen. Ventral und dorsalseitig zeigten sich am Rumpf scharf begrenzte Erytheme mit zungenförmigen Ausläufern und deutlicher Überwärmung, insgesamt circa 15 % der Körperoberfläche betreffend (Abb. 1). Abdominell imponierte das Erythem teilweise derb-bullös (Aspekt der *peau d’orange*) mit Einblutungen im Bereich der Blasen. Im Bereich der Erytheme ließ sich die Haut auf Verschiebedruck ablösen (Nikolski-I-Phänomen positiv). Die Schleimhäute waren nicht betroffen.

## Diagnostik

Die Vitalparameter zeigten sich normwertig. Laborchemisch imponierte eine Erhöhung der Leukozyten auf 10,4 Mrd/l (Referenzwert: 3,8–1,3 Mrd/l) sowie des CRP-Wertes auf 211 mg/l (Referenzwert: < 3 mg/l). Ferner zeigte sich neben einer Elektrolytentgleisung mit Hyponatriämie von 129 mmol/l (Referenzwert: 135–145 mmol/l) und Hypokaliämie von 3,2 mmol/l (Referenzwert: 3,5–5,0 mmol/l) eine Erhöhung des Kreatininwertes auf 2,25 mg/dl (Referenzwert: 0,7–1,3 mg/dl) im Sinne einer akuten Niereninsuffizienz. Blutkulturen ergaben nach 5 Tagen kein Wachstum anaerober oder aerober Bakterien. Zum Ausschluss eines Steven-Johnson-Syndroms (SJS) respektive einer toxischen epidermalen Nekrolyse (TEN) erfolgten Probebiopsien der Haut für Schnellschnitt, Histologie und direkte Immunfluoreszenz (DIF). Mittels Schnellschnitt konnte eine TEN ausgeschlossen werden. Es zeigte sich eine fokale subkorneale Spaltbildung und ein subepidermales Ödem mit beginnender Blasenbildung und dichter perivasaler superfizieller Dermatitis, passend zu einer Staphylokokken-assoziierten Infektion. Die DIF zeigte sich unauffällig. Der bakterielle Abstrich ergab den Nachweis Koagulase-negativer Staphylokokken. Es wurde schließlich unter Berücksichtigung der typischen klinischen Merkmale (akutes Auftreten des Erythems, begleitet von Fieber und Schüttelfrost) und des histopathologischen Befundes die Diagnose eines multilokulären bullösen Erysipels gestellt.

## Therapie und Verlauf

**Abb. 2:**
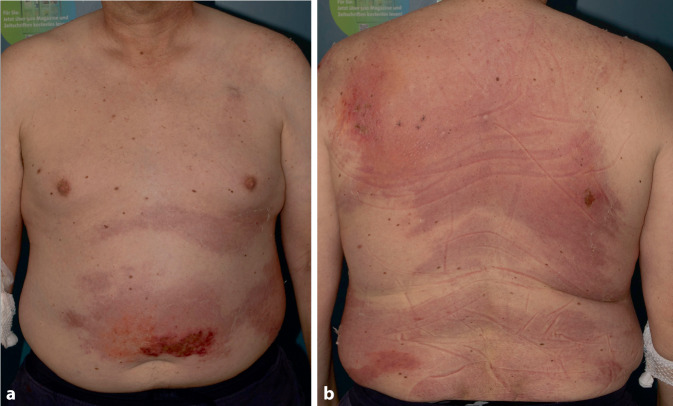
Verlaufsfoto nach 6‑tägiger antibiotischer Therapie: abblassendes Erythem (**a**: ventralseitig; **b**: dorsalseitig)

Es erfolgte aufgrund des stark eingeschränkten Allgemeinzustandes und der onkologischen Vorerkrankung zunächst eine internistische Führung und bei initial nicht eindeutiger Diagnose bei ungewöhnlicher Erythem-Manifestation eine kalkulierte intravenöse antibiotische Therapie mit Ampicillin (2000 mg) und Sulbactam (1000 mg) 3‑mal täglich. Nach Ausschluss konkurrierender Infektfoki erfolgte am Folgetag die dermatologische Übernahme. Hier zeigte sich der Patient bereits in deutlich stabilisiert, und die Entzündungsparameter waren bereits nach 2 Tagen deutlich regredient. Daher wurde sich für die Fortführung der etablierten antibiotischen Therapie für insgesamt 7 Tage entschieden und diese oral mit Amoxicillin (875 mg) und Clavulansäure (125 mg) 2‑mal täglich für weitere 7 Tage fortgeführt. Topisch erfolgte supportiv die Anwendung antiseptischer Umschläge. Darunter kam es zu einer Abheilung des bullösen Erythems (Abb. 2) und zu einem Rückgang der erhöhten Entzündungs- und Nierenretentionsparameter im Blut.

## Diskussion

Das Erysipel („erysipelas“, Wundrose) ist eine akute, bakterielle, nicht eiternde Infektion der oberen Dermis unter Beteiligung der Lymphspalten und -gefäße, zumeist bedingt durch β‑hämolysierende Streptokokken (Gram-positiv, Katalase-negativ) der Lancefield-Gruppe A (Synonym Streptococcus pyogenes) [1]. Darüber hinaus kommen, wie bei dem hier vorgestellten Patienten, auch bullöse Varianten vor, gegebenenfalls gefüllt mit serös-gelblichem, sterilen Inhalt und Einblutungen, ausgelöst durch bestimmte Virulenzfaktoren [1] und Mischinfektionen mit *Staphylococcus aureus* (Gram-positiv, Katalase-positiv, Koagulase-positiv; [2]). Das Erysipel ist typischerweise mit akut auftretendem Fieber und Allgemeinzustandsverschlechterung assoziiert [1]. Das charakteristische scharf begrenzte, flammenförmig anmutende Erythem (Rubor) mit leicht ödematös gespannter Haut [1] geht mit den weiteren Kardinalzeichen der Entzündung, Schmerz (Dolor), Überwärmung (Calor), Schwellung (Tumor) und Funktionsbeeinträchtigung (Functio laesa) einher. Die häufigsten Lokalisationen sind die untere Extremität oder das Gesicht, bedingt durch typische Eintrittsstellen für Bakterien, zum Beispiel die Onychomykose und Tinea pedis im Bereich der unteren Extremität [3]. Ein Auftreten am Rumpf hingegen ist äußerst selten, insbesondere bei Fehlen von chirurgischen Wunden oder sonstigen Eintrittspforten, wie beispielsweise Insektenstichen [4]. Bei dem hier präsentierten Patienten kommt als prädisponierender Risikofaktor für die ungewöhnliche Lokalisation des Erysipels ebenso wie für das multilokuläre Auftreten – in Form mehrerer unterbrochener Erytheme unterschiedlicher Rottöne – auch ein sekundäres Lymphödem nach Radiatio mediastinal und pulmonal in Betracht [1, 5]. Auch die bullöse Variante des Erysipels kann als besonders schwergradige Form mit gegebenenfalls langwierigen Verläufen eingestuft werden; gemäß Daten einer älteren Studie wird die Häufigkeit der bullösen Variante mit ca. 5 % angegeben [6]. Diese außergewöhnliche Präsentation kann die Diagnose des Erysipels erschweren; von besonderer Bedeutung ist daher das simultane Auftreten der Hautveränderungen mit akuter Allgemeinzustandsverschlechterung und Fieber. Die antiinfektive Therapie der Wahl ist Penicillin [1]. In Bezug auf das präsentierte Fallbeispiel muss angemerkt werden, dass bei der Diagnose Erysipel leitliniengerecht eine Therapie mit Penicillin, bei ausgeprägtem Befund gegebenenfalls unter Hinzunahme von Clindamycin für 3 Tage zur Unterdrückung der Toxinbildung, die erste Therapiewahl darstellt, da diese mit einer gezielteren Wirkung gegen Streptokokken und einem geringeren Risiko unerwünschter Ereignisse einhergeht [1, 7].

Von dem Erysipel abzugrenzen ist insbesondere die unkomplizierte (früher: begrenzte) Phlegmone, die meisten durch *Staphylococcus aureus* bedingt ist und die Dermis und Subkutis betrifft [1, 8]. Die unkomplizierte Phlegmone präsentiert sich typischerweise im Bereich einer Wunde als unscharf begrenztes, livides Erythem mit teigiger Schwellung. Insbesondere das ausgeprägtere Ödem, der lividere Farbton, die weniger scharfe Begrenzung, die unmittelbare Nähe zu einer Wunde und das Fehlen von Fieber zu Erkrankungsbeginn fungieren, wie auch in dem hier präsentierten Fallbeispiel, als wichtige Unterscheidungsmerkmale in der Abgrenzung zu dem Erysipel [8].

Die unkomplizierte Phlegmone wird mit Staphylokokken-wirksamen β‑Laktamantibiotika behandelt, zum Beispiel Cefadroxil oder Cefazolin. Komplizierte Phlegmonen betreffen auch tiefergehende Gewebe (Faszien oder Muskeln), gehen mit einem anderen Erregerspektrum (häufiger Anaerobier, gramnegative Bakterien, MRSA) einher und treten häufiger bei immunsupprimierten Patient:innen auf. Das klinische Bild ist gekennzeichnet durch ausgeprägtere Schwellung, Eiteransammlungen, Nekrosen und rasch progrediente Schmerzen [1, 8].

Als weitere Differenzialdiagnose im beschriebenen Fallbeispiel ist das „staphylococcal scalded skin syndrome“ (SSSS) zu nennen. Dieses wird durch exfoliative Toxine verursacht, die als Protease zur Spaltung von Desmoglein 1 und damit zur Ablösung der Haut führen [9]. Das SSSS tritt jedoch vor allem bei Säuglingen und Kleinkindern auf. Das Auftreten im Erwachsenenalter ist selten und betrifft in Regel Patienten mit Immundefizienz oder chronischer Niereninsuffizienz, einhergehend mit erhöhter Mortalität [9]. Klinisch präsentieren sich Betroffene in reduziertem Allgemeinzustand mit Fieber. Das Erythem manifestiert sich zu Beginn im Gesicht und ist rasch progredient. Das Nikolski-Zeichen ist positiv. Innerhalb von 24–48 Stunden kommt es zur Ausbildung großer, schlaffer Blasen, die schnell rupturieren und an Hautverbrennungen zweiten Grades erinnern.

Die wichtigste Differenzialdiagnose zum SSSS ist die TEN, eine medikamentös induzierte, lebensbedrohliche mukokutane Erkrankung mit Beteiligung der (Mund‑)Schleimhäute. Histologisch findet die Spaltbildung bei der TEN subepidermal statt, die Blasendecke besteht aus der gesamten eosinophilen nekrotischen Epidermis [10]. Das wichtigste klinische Unterscheidungsmerkmal zwischen SSSS und TEN ist die Schleimhautbeteiligung. Das SSSS geht typischerweise nicht mit Schleimhautbeteiligung einher und zeigt histopathologisch einen akantholytischen Spalt in der oberen Epidermis innerhalb des Stratum granulosum, entsprechend einer subkornealen Blase. Die Dermis ist frei von Entzündungszeichen. In der Akutsituation ist die Unterscheidung zwischen einer staphylokokkenassoziierten Erkrankung und einer TEN entscheidend und nur durch interdisziplinäre Zusammenarbeit und klinisch-pathologische Korrelation möglich.

Der hier dargestellte Fall zeigt eine ungewöhnliche Manifestation eines Erysipels am Rumpf mit zwei zusätzlichen seltenen klinischen Verlaufsformen (bullöses sowie multilokuläres Erysipel). Zusammenfassend möchten wir anhand des Fallbeispiels wichtige klinische Differenzialdiagnosen bei großflächigem Erythem mit bullöser Hautabhebung am Rumpf aufzeigen. Auch an ungewöhnlichen Lokalisationen sollte bei flammenförmigem Erythem das Erysipel in Betracht gezogen werden, insbesondere bei immunsupprimierten oder onkologisch vorerkrankten Patienten sowie bei bestehenden prädisponierenden Faktoren, wie sekundären Lymphödemen. Das begleitende Auftreten von Fieber und frühen Allgemeinsymptomen kann als wichtiges Unterscheidungskriterium zu anderen Differenzialdiagnosen, wie der unkomplizierten Phlegmone, dienen [1].

## Fazit für die Praxis

- Auch bei atypischer Lokalisation eines Erythems sollte bei immunsupprimierten oder onkologischen Patient:innen ein Erysipel in Betracht gezogen werden.
- Das Erysipel geht typischerweise mit deutlicher Allgemeinzustandsverschlechterung und plötzlich auftretendem Fieber einher, was die klinische Verdachtsdiagnose untermauert.
- Bei Erythem unklarer Ätiologie sollte die Diagnostik idealerweise durch eine Probebiopsie der Haut zur klinisch-pathologischen Korrelation ergänzt werden.
